# A pragmatic randomized controlled trial to improve inhaler technique using mHealth

**DOI:** 10.1186/s13601-020-00363-6

**Published:** 2020-12-07

**Authors:** Anna Vanoverschelde, Paulien van der Wel, Barbara Putman, Lies Lahousse

**Affiliations:** 1 Department of Bioanalysis, Pharmaceutical Care Unit, Ghent University, Ottergemsesteenweg 460, 9000 Ghent, Belgium; 2 Department of Medicine and Department of Environmental Medicine, New York University School of Medicine, New York, NY 10016 USA

## To the editor

Non-adherence and suboptimal inhaler technique are major problems in patients with obstructive lung disease. Less than one third of patients use their inhaler correctly, and the inhaler technique has not improved over the past 40 years despite innovations in devices [1]. Suboptimal inhaler use leads to more side effects, poor symptom control, reductions in health-related quality of life, exacerbations and consequently, increased healthcare costs [2, 3]. In order to achieve and maintain a correct inhaler technique, innovative approaches for education including mobile health applications (mHealth apps) are being explored [4]. The My Puff app, in which patients can choose their inhaler to watch instruction videos, could be an innovative approach to continue inhaler technique training between care visits. However, to date, the feasibility to integrate this app in patient care as well as the effectiveness of this app has not yet been evaluated. Therefore, we aimed to measure inhaler technique and disease control improvement three months after a pharmaceutical care intervention, comparing aid to install and use the My Puff app to providing standard information leaflets for continued home education.

This open randomized controlled trial was carried out between March and December 2018 in nine Belgian community pharmacies. Dutch-speaking adult patients with self-reported chronic asthma or Chronic Obstructive Pulmonary Disease (COPD) on registered inhaler therapy were included. Seventy patients were randomized to the app group (n = 37; 53%) or leaflet group (n = 33; 47%) with a randomized (computer-generated) block design per participating pharmacy. All patients were asked to demonstrate their inhaler technique, and received feedback as well as a demonstration of the correct technique with a similar placebo device. In order to continue inhaler training at home, patients in the control group received a standard leaflet with inhaler instructions developed by the Lung Foundation on behalf of the Lung Alliance Netherlands. Patients in the intervention group received maximum assistance to download and use the My Puff app (version 1.0.3–1.0.4), including a leaflet containing the Quick Response code of the app and guidance to install and use. My Puff is a free app with video-assisted inhaler instructions (≈ 3 min), developed by the Belgian Respiratory Society (BeRS) in 2017 (https://www.belgianrespiratorysociety.be/nl/mypuff). Patients’ inhaler technique was rated by the investigator using a checklist per device, and disease control was assessed by the Asthma Control Test® (ACT) and the COPD assessment Test® (CAT). A score increase of ≥ 3 for ACT and decrease by ≥ 2 for CAT was regarded as the minimum clinically important difference (MCID), and the potential impact of the intervention on this primary outcome was analyzed using a logistic regression model [5, 6]. Baseline characteristics of the 70 enrolled patients, using 133 inhalers in total, are presented in Table 1. According to the Global Initiative for Chronic Obstructive Lung Disease (GOLD) classification, 18% COPD/asthma-COPD overlap patients were classified GOLD A (n = 7), 49% patients GOLD B (n = 19), 3% patient GOLD C (n = 1) and 31% patients GOLD D (n = 12) based on the exacerbation history and CAT scores.

**Table 1:** Baseline demographic and clinical characteristics of the study patients

	Total (n = 70)	Application (n = 37)	Leaflet (n = 33)
Age in years, median (Q1–Q3)	64 (55–73)	66 (56–73)	63 (47–72)
Female, n (%)	39 (56)	20 (54)	19 (58)
BMI in kg/m², mean (SD)	26 (5)	26 (4)	27 (5)
Asthma, n (%)	31 (44)	18 (49)	13 (39)
COPD, n (%)	27 (39)	16 (43)	11 (33)
ACO, n (%)	12 (17)	3 (8)	9 (27)
Time since diagnosis in years, median (Q1–Q3)	13 (5–28)	15 (7–29)	10 (4–31)
Never smoker, n (%)	24 (34)	12 (32)	12 (36)
Past smoker, n (%)	32 (46)	18 (49)	14 (42)
Current smoker, n (%)	14 (20)	7 (19)	7 (21)
Pack-years among ever smokers, median (Q1-Q3)	30 (11–42)	31 (20–48)	26 (8–36)
Primary education, n (%)	11 (16)	4 (11)	7 (21)
Lower secondary education, n (%)	17 (24)	11 (30)	6 (18)
Upper secondary education, n (%)	22 (31)	14 (38)	8 (24)
Higher education (Non-university), n (%)	14 (20)	5 (14)	9 (27)
Higher education (University), n (%)	6 (9)	3 (8)	3 (9)
Allergy, n (%)	41 (59)	22 (59)	19 (58)
Chronic bronchitis, n (%)	37 (53)	21 (57)	16 (48)
Influenza vaccination, n (%)	44 (63)	22 (59)	22 (67)
≥ 1 exacerbation in preceding year, n (%)	34 (49)	12 (32)	22 (67)
≥ 1 severe exacerbation in preceding year, n (%)	8 (11)	4 (11)	4 (12)
≥ 1 course of antibiotics in preceding year, n (%)	46 (66)	21 (57)	25 (76)
≥ 1 course of oral corticosteroids in preceding year, n (%)	28 (40)	12 (32)	16 (48)
Handgrip strength dominant hand in kg, mean (SD)	30 (11)	31 (12)	29 (9)
1 inhaler, n (%)	24 (34)	15 (41)	9 (27)
2 inhalers, n (%)	31 (44)	13 (35)	18 (55)
3 inhalers, n (%)	13 (19)	9 (24)	4 (12)
4 inhalers, n (%)	2 (3)	0 (0)	2 (6)
DPI(s), n (%)	30 (43)	19 (51)	11 (33)
MDI(s), n (%)	8 (11)	3 (8)	5 (15)
SMI(s) (combinations), n (%)	11 (16)	3 (8)	8 (24)
DPI + MDI, n (%)	21 (30)	12 (32)	9 (27)
Single device, n (%)	38 (54)	22 (59)	16 (48)
SABA, n (%)	11 (16)	5 (14)	6 (18)
SABA/SAMA, n (%)	22 (31)	12 (32)	10 (30)
ICS, n (%)	2 (3)	1 (3)	1 (3)
LABA, n (%)	2 (3)	1 (3)	1 (3)
ICS/LABA, n (%)	36 (51)	19 (51)	17 (52)
LABA/LAMA, n (%)	7 (10)	5 (14)	2 (6)
ICS/LABA/LAMA, n (%)	23 (33)	11 (30)	12 (36)
Oral (leukotriene receptor antagonist or theophylline), n (%)	13 (19)	8 (22)	5 (15)
ACT score among asthma/ACO patients, median (Q1-Q3)	19 (13–23)	20 (14–24)	18 (13–22)
CAT score among COPD/ACO patients, median (Q1–Q3)	16 (10–22)	14 (10–21)	18 (11–24)

BMI, body mass index; COPD, chronic obstructive pulmonary disease; ACO, asthma-COPD overlap; DPI, dry powder inhaler; MDI, metered-dose inhalers; SMI, soft mist inhalers; SABA, short-acting β_2_-agonist, SAMA, short-acting muscarinic antagonist; ICS, inhaled corticosteroids; LABA, long-acting β_2_-agonist; LAMA, long-acting muscarinic antagonist; ACT, Asthma Control Test; CAT, COPD Assessment Test

**Fig. 1:**
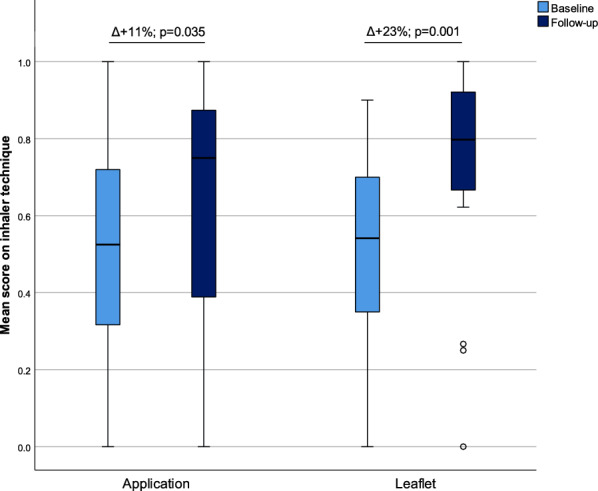
The mean score on inhaler technique at the start (t = 0 months) and the end of follow-up (t = 3 months; n = 60 patients with follow-up data)

The inhaler technique scored generally poor at baseline with half of patients making critical errors (n = 36, 51%). The mean inhaler technique score was significantly improved in both groups 3 months after the pharmacist intervention (Fig. 1), with no between-group differences in favor of the app over the leaflet for the continued home education (p = 0.116). In contrast, the proportion of patients achieving the MCID of improved disease control was 54% in the leaflet group and 28% in the app group resulting in a three times higher odds to achieve the MCID in the leaflet group compared to the app group (OR 2.95; 95% CI 1.01–8.60; p = 0.048). The mean age of the patients might have favored the leaflet. The positive effect on disease control seemed primarily driven by asthma control with an average 2 point improvement (p = 0.05). This is in line with inhaler technique interventions at community pharmacies demonstrating benefit for asthma control, although a consistent and important clinical benefit could not be observed in all studies [7]. Our study could not detect significant changes in disease control for COPD patients, which is in accordance with the pharmaceutical care intervention for COPD patients (PHARMACOP) study [8]. The pharmacist was not able to install the app on any of patients’ smartphones during the pharmacy visit due to the lack of a smartphone or code to the app store or mobile data. Since this pragmatic trial aimed to test the effectiveness of the app in daily practice, having a smartphone was no eligibility criterion [9]. Only a quarter of patients (n = 8) installed the app at home and only 13% of patients (n = 4) actively used it. In contrast, more than half of patients (n = 16, 57%) actively used the leaflet. Importantly, the users of continued home education made already less critical errors at baseline (n = 7/20, 35%). In contrast, non-users are likely to benefit most since they had a higher baseline error rate (n = 25/40, 63%). The most reported barrier for both the app and leaflet was not finding it necessary, but the app had an additional barrier of never using apps or a smartphone, although three third of the app users fully agreed that the app ensures how to use medication, and found that the app had a positive effect on disease control. Face-to-face training could be important to improve inhaler technique given mixed success of multi-media trainings at distance [7]. Despite the crucial aspect of inhaler training in asthma and COPD management, still 6% reported to not being instructed on their inhaler technique by any caregiver before. This disappointing result is almost unchanged in comparison with the PHARMACOP study in 2011 (7%), leaving room for further improvement [8]. Interestingly, females were more poorly controlled at baseline and seemed to have used any of the two approaches more, but the differences were not significant.

The small sample size might be a limitation of the study though continuation was not considered meaningful because of the limited use of the app in real life, even after maximally guided instructions. Still, a pre-post analysis showed a significant improved inhaler technique most likely driven by the hands-on training by the pharmacist. No spirometry results were available to confirm the diagnosis or to classify severity. However, this limitation supports the pragmatic framework. The major shortcomings in the use of the app highlight the need for further research to include features associated with improved adoption and adherence. eHealth literacy, motivation, and preferences of patients should be considered to achieve improvement of inhaler technique by personalized trainings at the community level. Our study results inquire at least as much interest in further improvement of inhaler technique trainings by health care providers as there is for app development to further decrease disease burden of patients with obstructive lung disease in real life practice.

## Data Availability

The datasets generated during and/or analyzed during the current study available from the corresponding author on reasonable request.
